# M1 Macrophage Extracellular Vesicles and TLR3 Agonist Nanoparticles Down‐Regulate Immunosuppression and Metastasis via AKT/TAM in Triple‐Negative Breast Cancer

**DOI:** 10.1002/mc.70003

**Published:** 2025-06-24

**Authors:** Shirley V. de Paiva Souza, Andreza Conceição Veras Aguiar, Elizabeth Costa S. de Albuquerque, Christina Eich, Luis J. Cruz, Pablo Lara, Carla Jorquera‐Cordero, Raelle Ferreira Gomes, Regina Célia Monteiro de Paula, Rosemayre S. Freire, de Araújo Júnior Raimundo Fernandes de AraújoJúnior

**Affiliations:** ^1^ Postgraduate Program in Health Science Federal University of Rio Grande do Norte (UFRN) Natal Brazil; ^2^ Inflammation and Cancer Research Laboratory, Department of Morphology Federal University of Rio Grande do Norte (UFRN) Natal Brazil; ^3^ Translational Nanobiomaterials and Imaging (TNI) Group, Department of Radiology Leiden University Medical Center Leiden The Netherlands; ^4^ Department of Orthopedics University Medical Center Utrecht Utrecht The Netherlands; ^5^ Department of Organic and Inorganic Chemistry Federal University of Ceará, UFC Fortaleza Brazil; ^6^ Department of Physics Federal University of Ceará Fortaleza Brazil

**Keywords:** exosomes, immunotherapy, nanomedicine, targeted immune response, TLR agonizts

## Abstract

Metastasis induced by tumor immune escape has been implicated as one of the factors contributing to the aggressiveness of triple‐negative breast cancer. Macrophage type 1‐derived extracellular vesicles were isolated and combined with PLGA nanoparticles loaded with the TLR3 agonist poly I:C as a therapeutic strategy to investigate their antitumor activity by downregulating tumor immune escape in the tumor microenvironment (TME) of breast cancer in a murine model of orthotopic tumor growth. Tumors were evaluated by qRT‐PCR and immunohistochemistry. Cellular uptake and polarization of murine macrophages (RAW 264.7 cells) were analyzed In Vitro by immunofluorescence and flow cytometry, respectively. Furthermore, mouse survival, lymph node involvement, and metastasis were also evaluated. In the animal model, the combination therapy inhibited tumor progression through TME immunomodulation, leading to a reduction in primary tumor size (*p* < 0.0001) and metastasis, along with an extension in survival of 11 days. Importantly, both innate and adaptive immune responses were enhanced, as indicated by increased CD8 expression (*p* < 0.0001) and reduced PD‐L1 levels in the TME, as well as elevated CD11c expression in lymph nodes (*p* < 0.0001). Likewise, the combination therapy suppressed tumor progression by reducing AKT1 expression (*p* < 0.001) and increasing E‐cadherin expression (*p* < 0.01). Based on these findings, the combination therapy functioned as a “vaccine‐like immunomodulatory strategy,” promoting TME immunomodulation and suppressing metastasis in a murine model of triple‐negative breast cancer.

**Abbreviations**
4T1: murine mammary carcinoma cell line
AKT1: protein kinase B 1
ANOVA: analysis of variance
ARG‐1: arginase 1
CCL2: chemokine (C‐C motif) ligand 2
CD: cluster of differentiation (CD68, CD163, CD8, CD11c, CD206)
CO₂: carbon dioxide
CSF‐1: colony‐stimulating factor 1
DAPI: 4’,6‐diamidino‐2‐phenylindole
DLS: dynamic light scattering
DMEM: Dulbecco's modified eagle medium
EMT: epithelial‐mesenchymal transition
EVs: extracellular vesicles
FACS: fluorescence‐activated cell sorting
FBS: fetal bovine serum
FEI: field emission instruments
HER2: human epidermal growth factor receptor 2
HRP: horseradish peroxidase
HSP70: Heat shock protein 70
IHC: immunohistochemistry
IL‐1: interleukin 1
IL‐10: interleukin 10
IL‐4: interleukin 4
M1EVs: type 1 macrophage‐derived extracellular vesicles
MHC II: major histocompatibility complex class II
mRNA: messenger RNA
NK: natural killer (cells)
NPIC: PLGA nanoparticles loaded with poly I:C
PBA: paraformaldehyde‐based fixative
PBS: phosphate‐buffered saline
PDI: polydispersity index
PD‐L1: programmed death‐ligand 1
PI: propidium iodide
PLGA: poly(lactic‐co‐glycolic acid)
PR: progesterone receptor
qRT‐PCR: quantitative reverse transcription polymerase chain reaction
RNA: ribonucleic acid
SEM: scanning electron microscopy
SYBR: SYBR Green
TAM: tumor‐associated macrophages
TGFβ: transforming growth factor beta
TLR3: toll‐like receptor 3
TME: tumor microenvironment
TNBC: triple‐negative breast cancer
TNF‐α: tumor necrosis factor alpha
ζ: zeta potential

## 1 Introduction

Triple‐negative breast cancer (TNBC) accounts for 10%–15% of new cases [1, 2]. In this cancer, there is no expression of estrogen receptors (ER), progesterone receptors (PR), or human epidermal growth factor receptor 2 (HER2), which impairs treatment efficacy and highlights the need for immunotherapies [3, 4, 5]. The complex interactions between immune cells and cancer cells in the tumor microenvironment (TME) promote strong immunosuppression, contributing to tumor invasion and metastasis [6, 7]. Additionally, this immunosuppressive environment alters tumor‐associated immune cells, such as tumor‐associated macrophages (TAMs) [8, 9].

In the TME, type 1 macrophages (M1) initially invade the tumor in an attempt to destroy tumor cells by secreting cytokines such as IL‐1 and TNF‐α, which activate CD8 + T lymphocytes and natural killer (NK) cells [10, 11]. However, tumor cells begin to release cytokines like IL‐10 and TGFβ, recruiting monocytes from the bone marrow and stimulating their differentiation into TAMs within the TME [12]. This process also involves the reprogramming of M1 macrophages into M2 macrophages, resulting in a pro‐tumor profile favoring the immune escape [13, 14].

However, immune cell populations within the TME are highly heterogeneous and cannot be strictly defined by single markers alone. TAMs can polarize into multiple subtypes, influenced by factors such as colony‐stimulating factor 1 (CSF‐1) and chemokine (C‐C motif) ligand 2 (CCL2), and often express M2‐like markers including CD206, CD163, Arginase 1 (ARG1), and IL‐10, all of which promote tumor progression. Moreover, CD163⁺ M2 TAMs are associated with elevated programmed death‐ligand 1 (PD‐L1) expression, further contributing to immunosuppression within the TME [15, 16, 17] C. In this context, many current therapies aim to stimulate M1 macrophages within tumors by reprogramming M2 phenotypes or recruiting macrophages to reduce immunosuppression in the TME [18, 19].

Furthermore, dysregulated signaling pathways drive tumor progression and invasion in the TME [20, 21, 22]. The protein kinase B 1 (AKT1) pathway, for instance, is frequently overexpressed in tumor cells and TAMs, promoting invasion, metastasis, and epithelial–mesenchymal transition (EMT) [23, 24, 25, 26]. EMT is marked by reduced E‐cadherin expression, a key adhesion molecule [27]. Thus, targeting signaling pathways in TAMs represents a promising strategy to modulate the TME and enhance antitumor immune responses [28].

Carriers with biocompatible and specific properties, such as extracellular vesicles, cellular fragments, and poly(lactic‐co‐glycolic acid) (PLGA) nanoparticles, hold significant promise in immunotherapy [29, 30]. Extracellular vesicles derived from M1 (M1EVs) have been indicated as a strategy for reprogramming TAMs and activating the immune system [31]. With an increased presence of M1 macrophages in the TME, the expression of immunosuppressive molecules such as PD‐L1 and the AKT pathway is reduced [32, 33].

In addition, toll‐like receptor 3 (TLR3) agonizts have been extensively investigated for treatment. These agonizts, when delivered by nanoparticles, activate TLR3, inducing cell death via endosomal TLR3 activation, and act as potential immunomodulators [34, 35]. In dendritic cells and macrophages, this stimulation facilitates activation, leading to CD8 + T cell activation against the tumor [36, 37, 38].

In this study, the association between type 1 macrophage‐derived extracellular vesicles (M1EVs) and poly(lactic‐co‐glycolic acid) (PLGA) nanoparticles loaded with the TLR3 agonist polyinosinic:polycytidylic acid (poly I:C) (NPIC) was analyzed to enhance the immune response of the TME in mice with TNBC.

## 2 Materials and Methods

### 2.1 Cell Culture

The macrophage cell line (RAW 264.7) and murine mammary carcinoma cells (4T1) were purchased from Banco de Celulares do Rio de Janeiro (BCRJ, Brazil). Dulbecco's Modified Eagle Medium (DMEM) supplemented with 10% fetal bovine serum and 1% antibiotic was used. Cells were maintained at 37°C in a humidified incubator with 5% CO₂.

### 2.2 Synthesis of M1EVs and Characterization of M1EVs

The nanosystems were synthesized at the Department of Radiology, University of Leiden. Extracellular vesicles (EVs) were obtained as previously described [31, 39], and purified using differential centrifugation followed by size‐exclusion [40]. The size and concentration of EVs were analyzed using a NanoSight NS300 (Malvern) at camera level 9 and detection threshold 3 [31]. Morphological characterization was performed by scanning electron microscopy (SEM) using a Quanta 450‐FEG–FEI microscope.

### 2.3 Synthesis and Characterization of PLGA Nanoparticles Loaded With Poly I:C

PLGA nanoparticles were synthesized using Resomer RG502H (Evonik Industries) via double‐emulsion solvent evaporation, as previously described [41]. Antigen release was quantified using the Pierce MicroBCA Protein Assay Kit (Thermo Fisher Scientific). Particle size and polydispersity index (PDI) were determined by dynamic light scattering (DLS) using a Zetasizer Nano ZSP with software version 7.12 (Malvern Panalytical). Zeta (ζ) potential was determined by laser Doppler velocimetry with particles dispersed in ultrapure water at 25°C, applying the Helmholtz–Smoluchowski model. Morphological analysis was performed similarly to that of M1EVs.

### 2.4 Polarization of RAW 264.7 Macrophages Cells Toward M2

RAW 264.7 cells were seeded at a density of 5 × 10⁵ cells per well in a 6‐well plate, supplemented: DMEM, 10% FBS and 1% antibiotic. Cells were incubated at 37°C and 5% CO₂ for 24 h, then treated with 0.04 µg/mL IL‐4 in serum‐free medium for 48 h. The expression levels of Arg‐1, CD206, CD163, and MHC II (Table S1) were evaluated to confirm macrophage polarization. Images were acquired at 400x magnification using a white light microscope (Axio Vert.A1, Carl Zeiss Suzhou Co. Ltd.). Total RNA was extracted using the TRIzol® reagent and purified with the SV Total RNA Isolation System. cDNA was synthesized using the High‐Capacity RNA‐to‐cDNA kit, and real‐time quantitative PCR was performed with the NZYSupreme qPCR Green Master Mix (2x). Experiments were conducted under standard qRT‐PCR conditions. Relative gene expression was calculated using the 2^ΔΔCt method.

### 2.5 Cell Uptake

To analyze the internalization of nanosystems in RAW 264.7 and RAW 264.7 + IL‐4 cells (polarized to the M2 profile), 2 × 10⁴ cells per well were plated in a 12‐well plate on round glass coverslips (12 mm) with DMEM supplemented with 10% FBS and 1% antibiotic. The treatments were stained 24 h prior with propidium iodide (PI ‐ 100 μg/mL), continuous agitation and protected from light [42].

### 2.6 Flow Cytometry

RAW 264.7 cells were plated at 2 × 10⁵ cells per well in 6‐well plates with DMEM, 10% FBS and 1% antibiotic. After 24 h, the medium was removed and divided into the following groups: (1) Unlabeled; (2) M1EVs; (3) NPIC 0.125 mg/mL; (4) M1EVs + NPIC 0.125 mg/mL. Serum‐free DMEM and the cytokine IL‐4 were added. After 48 h, the cells were detached and washed with 1× Phosphate‐Buffered Saline (PBS), centrifuged at 2500 rpm for 5 min. Then, 0.5% PBA was applied for 45 min at room temperature. The tubes were centrifuged and anti‐CD68 (Cat# MA1‐82739; 1:500; Invitrogen) and anti‐CD163 (Cat# 46‐1631‐80; 1:500; Invitrogen) antibodies were added to the FACS tubes. The analysis was performed on the BD FACSCanto II flow cytometer, and the data were analyzed using FlowJo software.

### 2.7 In Vivo Assays

#### 2.7.1 Animals

Female Balb/c mice 7–8 weeks, 20–25 g, were used for the animal experiment and were obtained from the Keizo Asami Immunology Laboratory (FIOCRUZ‐PE). The animals were housed under standard conditions with ad libitum access to food and water and were handled in accordance with procedures approved by the Ethics Committee of the Federal University of Rio Grande do Norte (No. 222.011/2020).

### 2.8 Orthotopic Breast Cancer Model With Survival

To induce triple‐negative breast cancer, 1 × 10⁶ 4T1 cells were injected subcutaneously into the left fourth mammary gland of the female mice [42]. When the tumor reached 3 mm, the animals (5 per group) were divided: (1) Saline; (2) M1EVs; (3) NPIC; (4) M1EVs + NPIC. Three treatments were administered every 5 days. The animals were monitored and weight and tumor size were measured. After 21 days, the animals were observed daily. The saline group was euthanized at 28 days due to endpoint criteria such as reduced mobility. Mice from the other groups were observed for more than 28 days to assess survival over different timelines. Tumor volume was calculated using the formula: mm³ = (width × length²) × 0.52 [31].

The animals were euthanized with 80 mg/kg ketamine and 20 mg/kg xylazine. The tumors, liver, lung, axillary lymph nodes and inguinal lymph nodes, were collected. Half of each tumor was stored at −80°C for qRT‐PCR analysis, and the other half was placed in 10% paraformaldehyde for histopathological analysis. Blood samples were collected in EDTA‐containing tubes for red blood cell and hematocrit analysis [42].

### 2.9 Tissue Preparation and Histopathological Analysis

For the histological analyses, liver, lung, inguinal lymph node, and tumor tissues were fixed in 10% paraformaldehyde and subsequently underwent histological processing. The liver and lung slides were analyzed as previously described to semi‐quantitatively assess metastatic foci [42]. Additionally, a semiquantitative scoring system based on previous studies was applied to evaluate these organs [43]. Pulmonary parameters were evaluated using a score ranging from 1 to 4, where 1 = absent, 2 = mild, 3 = moderate, and 4 = severe, fields were evaluated at 40× magnification and scored based on morphological criteria adapted from a previous study [44]. A trained examiner analyzed images at 100× and 400× magnification using a light microscope (Nikon Eclipse 2000 equipped with Nikon DS‐Fi2; Nikon Corporation, Tokyo, Japan).

### 2.10 Immunohistochemistry

For immunohistochemistry, three samples from each group were selected for the tumor and the inguinal lymph node. The tissues were placed on silanized slides. After antigen retrieval, the tumor sections were incubated with the primary antibodies: anti‐PDL1, anti‐AKT, anti‐E‐cadherin, and anti‐CD8a overnight at 4°C. The lymph node tissue was incubated with anti‐CD11c. Secondary goat anti‐mouse antibody was used for anti‐AKT, anti‐CD8a, and anti‐CD11c overnight at 4°C. Polink2 HRP polymer detection system was used for anti‐E‐cadherin and anti‐PDL1 (reagents‐Table S3). Diaminobenzidine was applied followed by hematoxylin counterstaining. Images were captured using a light microscope at 400× magnification (Nikon Eclipse 2000 equipped with Nikon DS‐Fi2; Nikon Corporation, Tokyo, Japan). The analysis followed a previously described scoring system [45].

### 2.11 Tumor and Lymph Node Analysis by qRT‐PCR

The tissues, stored at −80°C in Trizol reagent (Invitrogen), were homogenized and processed using the ethanol/chloroform extraction method. Total RNA was isolated using the SV Total RNA Isolation System (Promega). After quantification and normalization to 70 ng/μL, cDNA was synthesized using the High Capacity cDNA Reverse Transcription Kit (Cat# 2849352, Invitrogen). For labeling, a SYBR Green‐like dye (Cat# A6001, GoTaq qPCR master mix, Invitrogen) was used and standard conditions were applied. Gene expression was normalized to the reference gene β‐actin using the 2^(−ΔΔCt) method. The primers used are listed in Table S2.

### 2.12 Statistical Analysis

The statistical tests applied included one‐way ANOVA followed by Bonferroni post hoc correction. Flow cytometry and uptake assays were conducted in duplicates, all other assays in triplicate. Statistical significance was defined as p‐values less than 0.05 (*p < 0.05, **p < 0.01, ***p < 0.001, and ****p < 0.0001) and GraphPad Prism 9.0 software (La Jolla, CA, USA) was used for analysis.

## 3 Results

### 3.1 Characterization of Nanosystems

**Figure 1:**
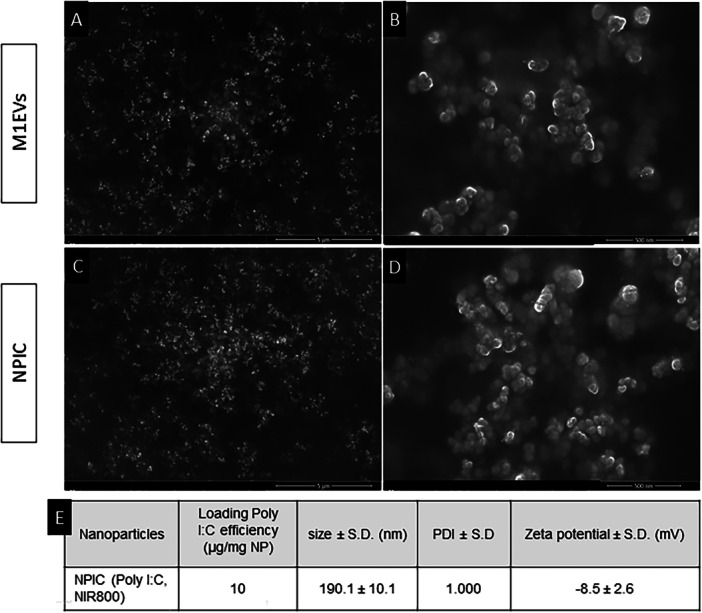
Characterization of M1EVs and NPIC. Scanning electron microscopy (SEM) images of M1EVs (A, B) at 25,000× (A) and 200,000× (B) and NPIC (C, D) at 25,000× (C) and 200,000× (D). (E) NPIC data: a poly I:C loading efficiency of 10 μg/mg, size of 190.1 ± 10.1 nm, with PDI of 1.000 and zeta potential of −8.5 ± 2.6 mV. Scale bar: 5 μM (A, C) and 500 nm (B, D).

Morphological characterization was performed using SEM (Figure 1A–D), along with measurements of size, zeta potential, and polydispersity index (PDI) for NPIC and M1EVs (Figure 1E). The loading efficiency of Poly I:C in NPIC was determined to be 10 µg/mg of nanoparticles (Figure 1E). NPIC exhibited a mean size of 190.1 ± 10.1 nm, a PDI of 1.000, and a zeta potential of −8.5 ± 2.6 mV. The M1EVs had been previously characterized, with a size range of 100–200 nm and the expression of specific M1EV markers, integrin A6 and HSP70, confirmed by western blot [31, 39].

### 3.2 Polarization of RAW 264.7 Macrophage Cells Toward M2

Macrophage polarization efficiency was evaluated through morphological assessment (Figure S1A,B) and M2 marker expression (ARG‐1, CD206, CD163, and MHC II) by qRT‐PCR (Figure S1C). Figure S1A shows rounded, oval‐shaped RAW 264.7 cells growing in clusters. In contrast, Figure S1B reveals that RAW 264.7 cells + IL‐4 exhibit cytoplasmic extensions and occupy more surface area. Gene expression analysis indicated significantly higher mRNA levels of ARG‐1 (*p* < 0.001), CD206, and CD163 (both *p* < 0.0001) in RAW 264.7 + IL‐4 cells compared to untreated controls. No significant difference was observed in MHC II expression between groups.

### 3.3 The Efficiency of Nanoparticle Internalization

**Figure 2:**
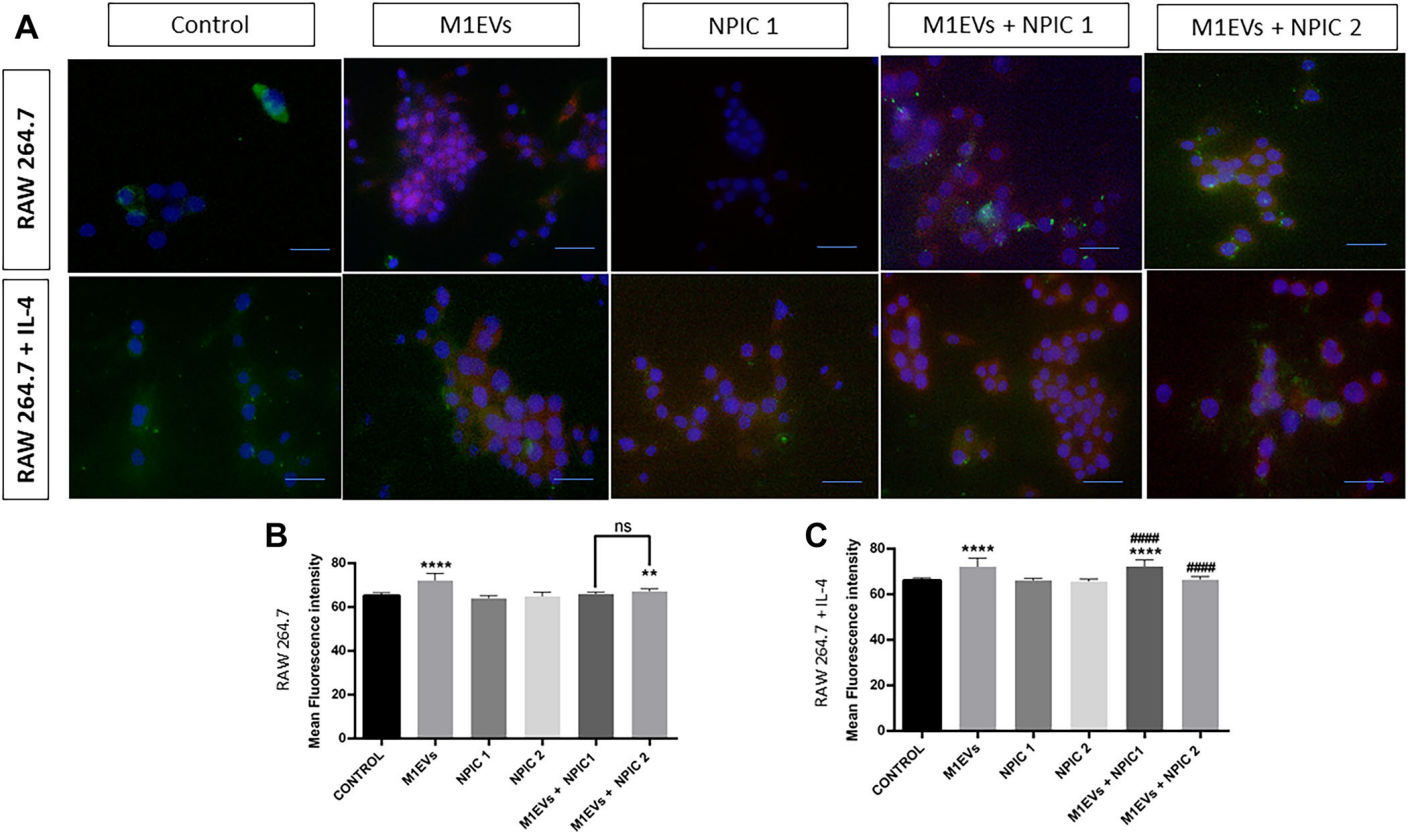
Internalization of treatments by RAW 264.7 and RAW 264.7 + IL‐4 cells. Nuclei are stained blue with DAPI, cell membranes green with DiO, and propidium iodide (red) indicates the treatment. Images A show RAW 264.7 cells: control, M1EVs, NPIC 1 (0.125 mg/mL), M1EVs + NPIC 1, M1EVs + NPIC 2 and RAW 264.7 + IL‐4 cells: control, M1EVs, NPIC 1 (0.125 mg/mL), M1EVs + NPIC1, M1EVs + NPIC 2. Graphs B (RAW 264.7) and C (RAW 264.7 + IL‐4) show the mean fluorescence intensity cells. No significance (ns); Data are shown as the mean ± SD (*p < 0.01, ***p < 0.0001 vs. control; *####p* < 0.0001 vs. M1EVs). Scale bar: 50 µM.

Figure 2A, shows representative cell images: nuclei stained blue (DAPI), membranes green (DiO), and nanoparticles red (propidium iodide). Statistical analysis (Figure 2B) revealed that RAW 264.7 cells treated with M1EVs demonstrated significantly greater internalization than untreated controls (*p* < 0.0001). The combination of M1EVs + NPIC 2 (0.25 mg/mL) also showed increased uptake (*p* < 0.01). No significant difference was found between NPIC 1 and NPIC 2 groups. In RAW 264.7 + IL‐4 cells, M1EVs also showed enhanced internalization (*p* < 0.0001). Notably, only M1EVs + NPIC 1 (0.125 mg/mL) showed significant uptake compared to the control (*p* < 0.0001). Furthermore, both M1EVs + NPIC 1 and NPIC 2 significantly outperformed M1EVs alone (*p* < 0.0001). Based on these results, M1EVs + NPIC 1 was selected for further In Vitro assays.

### 3.4 Flow Cytometry Analysis of CD68 Expression

**Figure 3:**
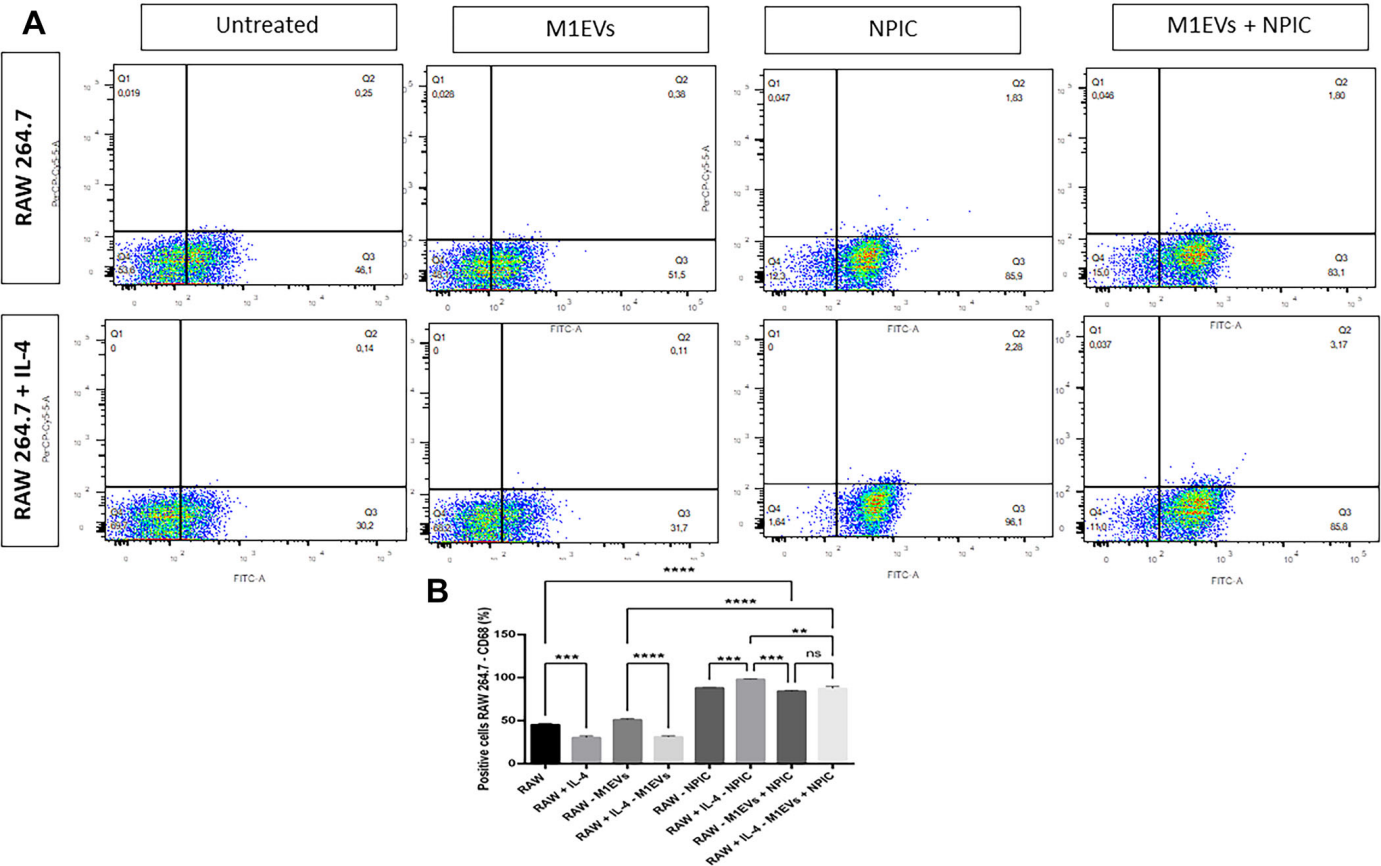
Flow cytometry analysis of treatment marked CD68, by RAW 264.7 and RAW 264.7 + IL‐4 cells. Image A shows RAW 264.7 cells: untreated, M1EVs, NPIC, M1EVs + NPIC; and RAW 264.7 + IL‐4 cells: untreated, M1EVs, NPIC, M1EVs + NPIC. Graph B presents the percentage of positive cells marked with CD68, for each treatment group. Method: two‐way ANOVA with post hoc Kruskal–Wallis. No significance (ns); Data are shown as the mean ± SD (*p < 0.01, ***p < 0.001, ***p < 0.0001 compared to untreated and between groups).

CD68 is the marker to evaluate the increase in the population of cells with a type 1 macrophage profile. Its expression was assessed by flow cytometry in RAW 264.7 cells and RAW 264.7 + IL‐4 cells (Figure 3A), along with a statistical comparison between these groups and the corresponding treatment conditions (Figure 3B). Statistical analysis revealed a significant increase in CD68 expression in RAW 264.7 cells (*p* < 0.001) compared to RAW 264.7 + IL‐4 cells. Additionally, in RAW 264.7 cells, CD68 expression was significantly higher following treatment with M1EVs compared to RAW 264.7 + IL‐4 cells (*p* < 0.0001). In the group treated with M1EVs + NPIC, a further increase in CD68 expression was observed (*p* < 0.0001) compared to untreated RAW 264.7 cells and those treated with M1EVs alone. Similarly, in RAW 264.7 + IL‐4 cells, the combination of M1EVs + NPIC led to higher CD68 expression compared to RAW 264.7 + IL‐4 cells alone (*p* < 0.0001).

### 3.5 Analysis of CD163 Expression By Flow Cytometry

CD163 expression, indicative of the M2 phenotype, was assessed in RAW 264.7 and RAW 264.7 + IL‐4 cells (Figure S2A,B). Statistical analysis (Figure S2B) revealed that both cell types exhibited an increase in CD163 expression (*p* < 0.0001) compared to their respective controls, primarily due to the effect of NPIC, which was also observed in the M1EVs + NPIC combination. Additionally, it was noted that RAW 264.7 cells treated with M1EVs + NP115 showed a reduction in CD163 expression (*p* < 0.0001) when compared to RAW 264.7 cells treated with NPIC alone, indicating that the combination of treatments In Vitro can reduce M2 polarization in undifferentiated cells.

### 3.6 In Vivo Study

**Figure 4:**
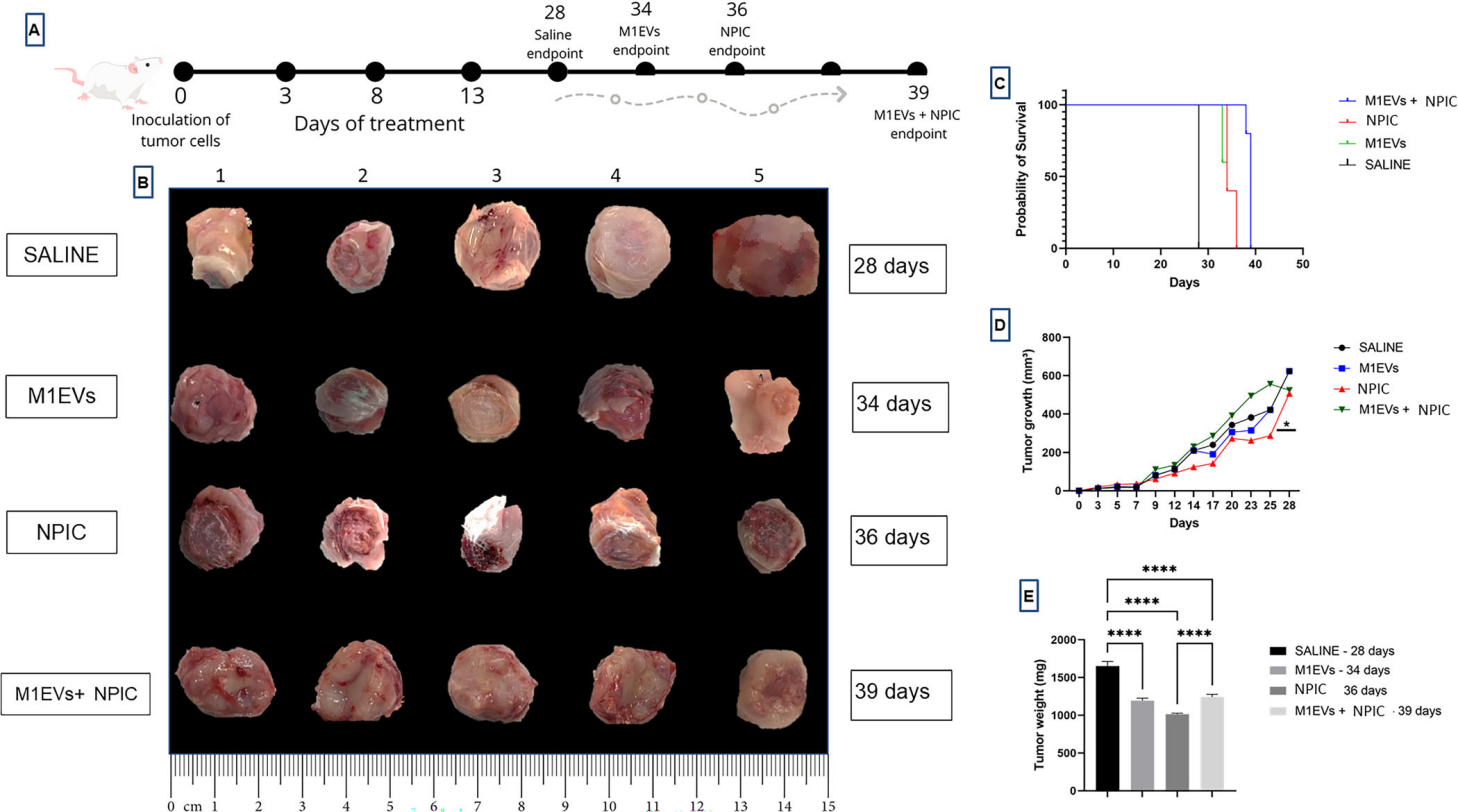
Orthotopic model of breast cancer. In illustration A, the entire process is shown (Day 0), the days of treatments (Days 3, 8, and 13), and the average period of animal euthanasia, which varied among each group. In illustration B, tumors from each of the five animals per group (1–5) are shown, with each line representing one group: saline, M1EVs, NPIC, and M1EVs + NPIC. In graph C, the probability of survival of the animals is observed. In graph D, the tumor growth curve of each group is depicted. In graph E, the tumor weights are presented. No significance (ns); Data are shown as the mean ± SD (*p < 0.05, ****p < 0.0001 vs. untreated; ***p < 0.001, ***p < 0.0001 comparing specific groups).

Subcutaneous tumors were photographed across treatment groups: Saline (28 days), M1EVs (34 days), NPIC (36 days), and M1EVs + NPIC (39 days) (Figure 4B). The combination group had the longest survival. The full experimental workflow is illustrated in Figure 4A. Survival curves (Figure 4C) show that the M1EVs + NPIC group had the highest survival rate, followed by NPIC, with saline showing the lowest. Tumor volume over 28 days (Figure 4D) revealed that NPIC‐treated animals had significantly smaller tumors (*p* < 0.05). Final tumor weights (Figure 4E) were significantly lower in all treated groups compared to saline (*p* < 0.0001). Table S4 presents red blood cell and hematocrit levels, which remained within normal ranges.

### 3.7 Immunohistochemistry of Markers to Tumor Progression, Metastasis, and Immune Activation

**Figure 5:**
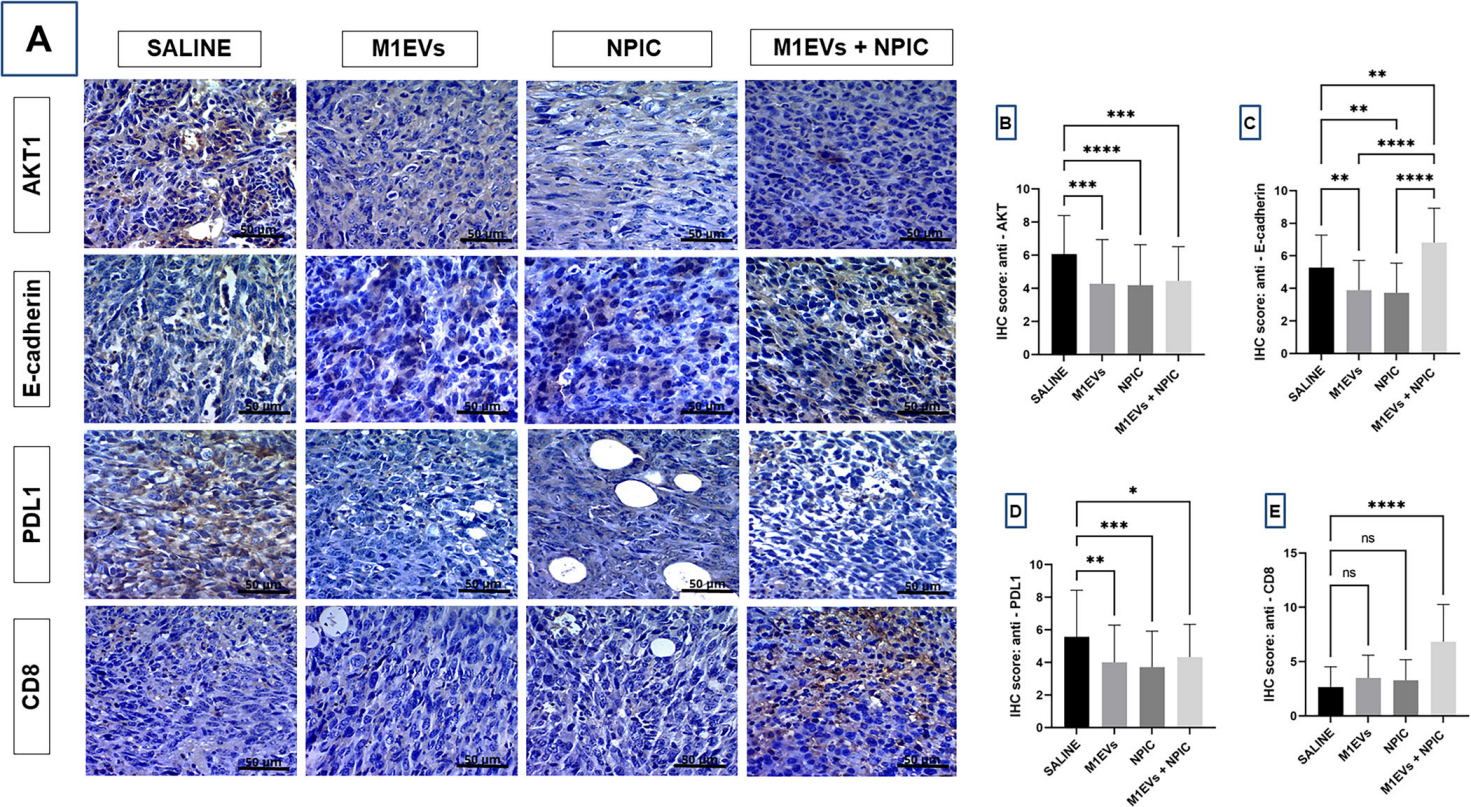
Tumor fragment immunohistochemistry panel. Image (A) AKT1, E‐cadherin, PD‐L1 and CD8 from the saline, M1EVs, NPIC and M1EVs + NPIC groups, identified in each column (400×). The statistical analysis of the score, which relates prevalence and marking intensity, of each marker is shown in the graphs: (B) AKT1; (C) E‐Cadherin; (D) PD‐L1 and (E) CD8. Statistical analysis was performed using ANOVA followed by Bonferroni posttest. No significance (ns); Data are shown as the mean ± SD (*p < 0.05, **p < 0.01, ***p < 0.001, ****p < 0.0001 vs. saline group).

**Figure 6:**
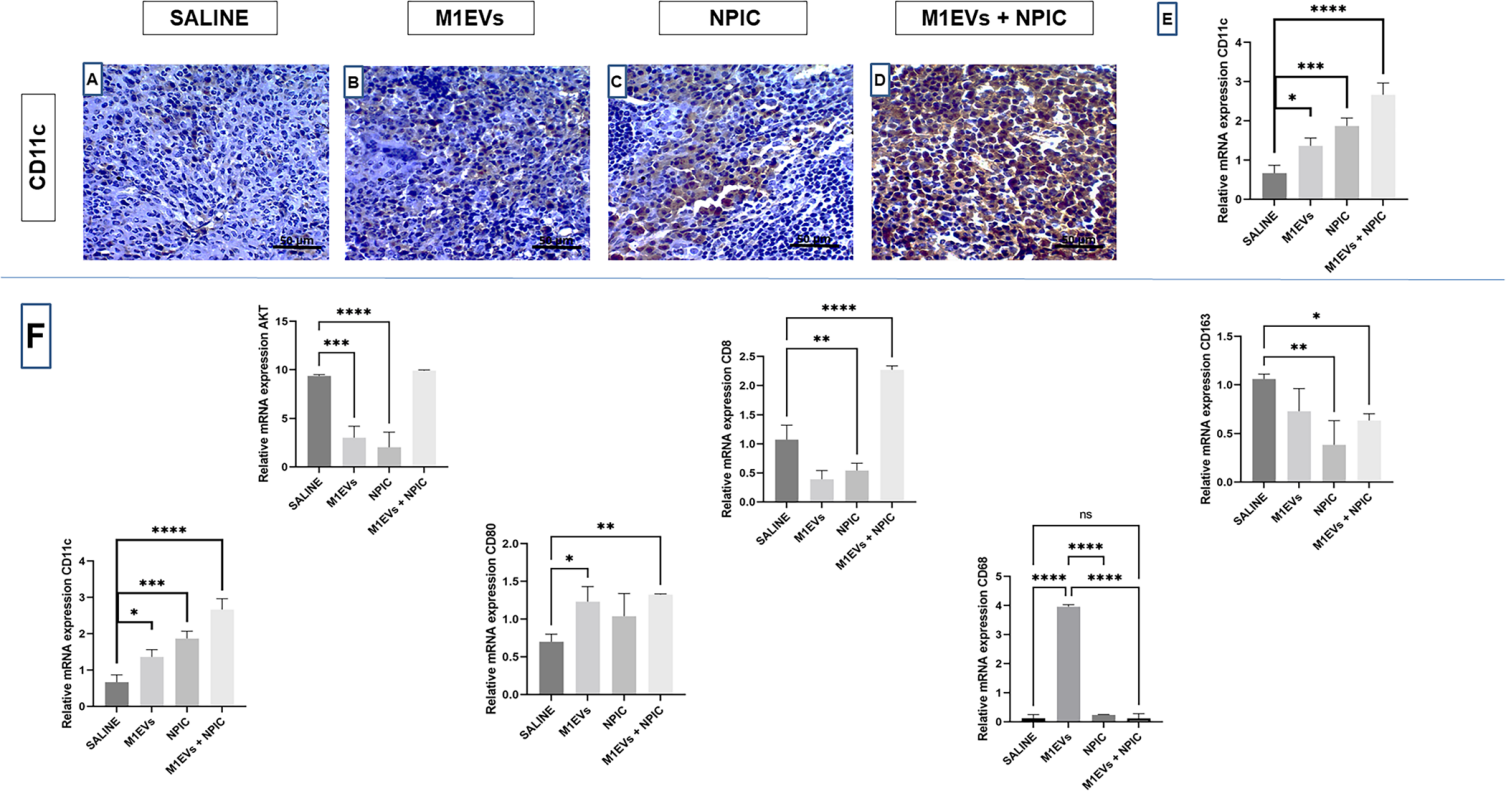
Immunohistochemistry of the lymph node and relative mRNA expression of the tumors. The first section (lymph node) of the image shows the immunohistochemistry of the CD11c marker, of dendritic cells in the groups: saline solution (A), M1EVs (B), NPIC (C) and M1EVs + NPIC (D). In addition to graph (E), which shows the statistical analysis of immunostaining through the score. In the second part (tumors) of the figure–(F), below the blue line, the markers AKT, CD8, CD11c, CD80, CD68 and CD163 are seen. Statistical analysis was performed using ANOVA followed by Bonferroni posttest. No significance (ns); Data are shown as the mean ± SD (*p < 0.05, **p < 0.01, ***p < 0.001, ***p < 0.0001).

Immunohistochemical analysis (Figure 5) for the markers AKT1, E‐cadherin, PD‐L1, and CD8 was compared among the groups: Saline, M1EVs, NPIC, and M1EVs + NPIC. The saline exhibited high expression levels of AKT1 and PD‐L1 (Figure 5B,D), indicating possible immune evasion. In the treated groups, particularly the M1EVs + NPIC group, a significant reduction in the expression of AKT1 (*p* < 0.001) (Figure 5B) and PD‐L1 (*p* < 0.05) (Figure 5D) was observed, suggesting diminished immune suppression. Expression of E‐cadherin was higher (*p* < 0.01) in the M1EVs + NPIC group compared to saline (Figure 5C), indicating the preservation of cell adhesion, even after 11 additional days. Expression of CD8 was significantly increased in the M1EVs + NPIC group (*p* < 0.0001) (Figure 5E), highlighting a more robust immune response. Lymph node analysis revealed increased CD11c expression (*p* < 0.0001) in the combination group (Figure 6A–E), highlighting enhanced dendritic cell recruitment.

### 3.8 Gene Expression of Markers Related to Immunomodulation by qRT‐PCR

Gene expression in tumor tissue (Figure 6F) showed decreased AKT levels in the M1EVs (*p* < 0.001) and NPIC (*p* < 0.0001) groups compared to saline. CD8, a marker of cytotoxic cells, an increase was observed in the NPIC (*p* < 0.01) and M1EVs + NPIC (*p* < 0.0001) groups compared to the control. For another marker of immune activity, specifically for dendritic cells: CD11c+ , higher expression was observed in tumors across all treated groups, particularly in the M1EVs + NPIC combination (*p* < 0.0001). Regarding CD80 (Figure 6F), a marker of antigen‐presenting cells (macrophages, B lymphocytes, and dendritic cells), an increase in expression was noted when compared to saline, specifically in M1EVs (*p* < 0.05) and M1EVs + NPIC (*p* < 0.01). CD68 was elevated only in M1EVs (*p* < 0.0001). CD163 was significantly downregulated in the NPIC (*p* < 0.01) and M1EVs + NPIC (*p* < 0.05) groups.

### 3.9 Histopathological Analysis

Histopathological analysis in the liver and lung (Figure S3A) revealed notable differences in the quantity of metastatic foci among the treatment groups: Saline, M1EVs, NPIC, and M1EVs + NPIC. In liver sections, the saline group exhibited metastatic foci showing high cellular density. In contrast, the M1EVs and NPIC groups demonstrated a marked reduction in the number of metastatic foci (*p* < 0.01) (Figure S3B), although some tissue disorganization remained. Notably, the M1EVs + NPIC group displayed the lowest number of metastatic foci (*p* < 0.01) compared to the control. In lung sections (Figure S3C), the control group also exhibited multiple metastatic foci with increased cellular density.

**Figure 7:**
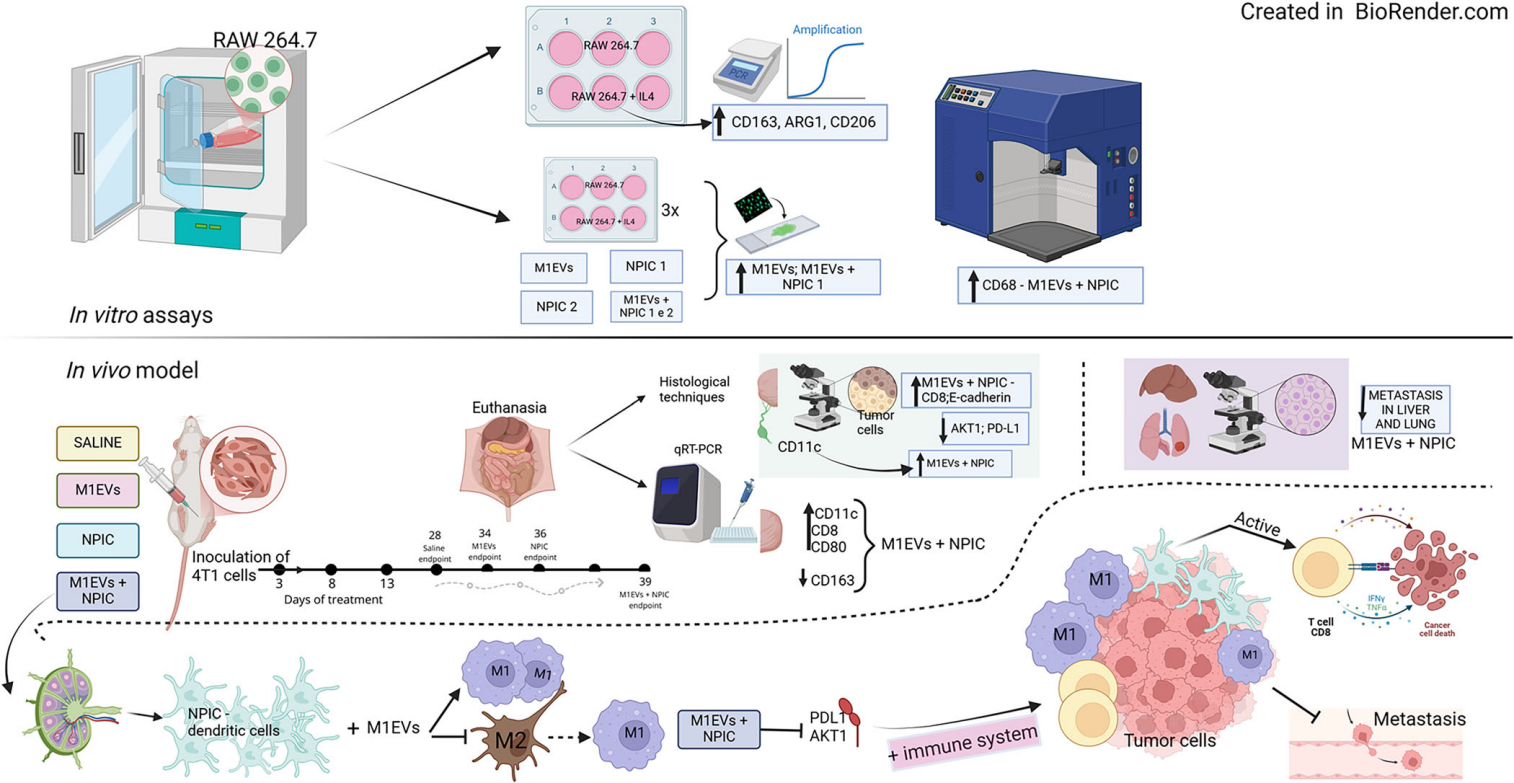
The illustration highlights the immunomodulatory effects of treatments in the tumor microenvironment of TNBC.

The M1EVs and NPIC groups showed similar areas of neoplastic cell infiltration. However, the M1EVs + NPIC group (which had an additional 11 days of survival compared to the control) (Figure S3C) demonstrated a significant reduction in metastatic niches (*p* < 0.01) compared to the saline group. Toxicity evaluation (Figure S4) revealed reduced liver fibrosis and necrosis in the M1EVs + NPIC group (*p* < 0.0001), and NPIC treatment reduced pulmonary hemorrhage and vascular congestion (*p* < 0.01). No severe organ toxicity was observed. Figure 7 provides a summary of the findings of this study.

## 4 Discussion

Immunotherapy with adjuvants plays a crucial role in the treatment of aggressive cancers, particularly in TNBC [46, 47, 48]. This study explored the isolated and combined treatments of vesicles derived from type 1 macrophages and PLGA nanoparticles containing the TLR3 agonist poly I:C to understand the mechanisms of immunomodulation in the TME.

M1EVs and NPIC were successfully characterized, showing suitable nanometric sizes for biomedical use [49]. M1EVs are fragments derived from type 1 macrophage cells, while NPIC is a PLGA nanoparticle, a material widely recognized as an effective drug carrier and adjuvant delivery system [50, 51, 52]. In this sense, M1EVs were efficiently internalized by M2‐polarized TAMs and this uptake was potentialized by combination with 0.125 mg/mL NPIC. It's known that the effectiveness of extracellular vesicles in intracellular delivery is due to their membrane composition which facilitates the uptake of other adjuvant compounds [53, 54, 55].

The CD68 expression, a marker of M1 macrophages, significantly increased in RAW 264.7 cells and M2‐polarized TAMs treated with M1EVs and M1EVs + NPIC combination. These results demonstrated the treatment ability to reprogram cells from an M2 to a pro‐inflammatory M1 profile [31, 39, 56]. Additionally, the combination of M1EVs + NPIC slightly reduced CD163 expression compared to NPIC alone in RAW 264.7 cells. As NPIC is not specific to a particular macrophage type, it may stimulate undifferentiated macrophages towards either an M1 or M2 profile, but association with M1EVs helped to enhance the antitumor M1 polarization while reducing protumor M2 polarization [31, 39, 57, 58].

In the In Vivo model, the combination therapy significantly reduced AKT1 and PD‐L1 expression in the TME, contributing to inhibition of tumor progression and immune evasion. AKT signaling, commonly upregulated in TNBC, promotes tumor growth and survival, while its reduction in treated tissues positively modulates the immune system [33, 59]. PD‐L1, which inhibits immune cell activation, was also less expressed in treated groups, correlating with increased CD8 + T cells in the TME [60, 61]. This increase in CD8+ cells, associated with improved overall survival, indicates enhanced cytotoxic T cell activation, contributing to improved immune responses in the treated animals [38, 62].

Additionally, there was increased expression of the protein CD11c, a dendritic cell marker, in both lymph nodes and tumors of the M1EVs + NPIC group. Dendritic cells are key antigen‐presenting cells in the innate immunity that activate the adaptive immune system via CD8 cells [63, 64]. This underscores NPIC's role in activating these cells and the ability of M1EVs to shift macrophages toward the M1 phenotype. For a more comprehensive functional profile, additional markers such as MHC‐II and CD86 are recommended for characterizing dendritic cell activation.

In the TME, cell proliferation, survival, and migration are regulated by the AKT signaling pathway, which was downregulated following treatment with M1EVs and NPIC [65]. This modulation is critical for restoring antigen presentation and promoting immune activation. The upregulation of CD80, a marker of antigen‐presenting cells (macrophages, B lymphocytes, and dendritic cells) that is essential for modulating immunosuppression, as it indicates the activation of CD8⁺ T cells and a functional shift of innate immune cells toward enhanced immunostimulatory activity, in the M1EVs and M1EVs + NPIC groups, along with the selective increase of CD68 in M1EVs, indicates a shift toward a pro‐inflammatory TME and antitumoral macrophage profile [66, 67, 68].

In parallel, the marked reduction in CD163 expression, particularly in the NPIC and M1EVs + NPIC groups, reflects a decrease in immunosuppressive TAMs, which are known to facilitate tumor progression [69, 70]. These findings reinforce the role of the AKT/TAM axis as a central mechanism modulated by the combined treatment.

Clinically, the M1EVs + NPIC combination extended survival by 11 days and significantly reduced tumor burden and metastasis, outperforming the monotherapies. This group also showed fewer metastatic foci in the lungs and liver, accompanied by elevated E‐cadherin expression, suggesting decreased metastatic potential [71, 72]. AKT activation suppresses E‐cadherin via SNAIL, promoting EMT and β‐catenin‐mediated Wnt signaling, which enhances invasion and metastasis. The group with combined treatment reduced AKT and increased E‐cadherin, aligning with the observed reduction in metastatic niches [73, 74]. Importantly, red blood cell counts and hematocrit levels remained within physiological ranges [75], indicating no systemic toxicity. This was corroborated by histopathological analysis of liver and lung tissues, which revealed no significant morphological alterations [39, 76].

In summary, the combined use of M1EVs and NPIC represents a promising immunotherapeutic strategy for modulating the immune system and controlling tumor growth. These findings are significant for the development of nanosystem‐based immunotherapies as vaccine‐like immunomodulatory strategies in triple‐negative breast cancer.

## Author Contributions

Shirley V. de Paiva Souza contributed to the experimental techniques, writing, and structuring of the manuscript. Christina Eich and Luiz J. Cruz formulated and characterized the NPIC, while Carla Jorquera and Pablo Lara formulated and characterized the M1EVs. Andreza Conceição Veras Aguiar and Elizabeth Costa S. de Albuquerque assisted with In Vitro techniques. Raelle Ferreira Gomes, Rosemayre S. Freire and Regina Célia Monteiro de Paula contributed to the morphological analysis of the nanosystems. Raimundo FA Júnior coordinated the experiments and designed this study.

## Ethics Statement

Animal Studies: The animals subjected are listed and approved by the ethics committee of the Federal University of Rio Grande do Norte (N°. 222.011/2020).

## Consent

The authors have nothing to report.

## Conflicts of Interest

The authors declare no conflicts of interest.

## Supporting information

Figure S1 1.

Figure S2 1.

Figure S3 1.

Figure S4.

Supporting Information revised.

## Data Availability

The data that support the findings of this study are openly available in Ex‐Shi at https://drive.google.com/drive/u/2/folders/.
