# Supplementary figures and images for: M1 Macrophage Extracellular Vesicles and TLR3 Agonist Nanoparticles Down‐Regulate Immunosuppression and Metastasis via AKT/TAM in Triple‐Negative Breast Cancer
Source: Mol Carcinog. 2025 Jun 24;64(9):1450–61. doi: 10.1002/mc.70003 (PMC12370002; doi:10.1002/mc.70003)

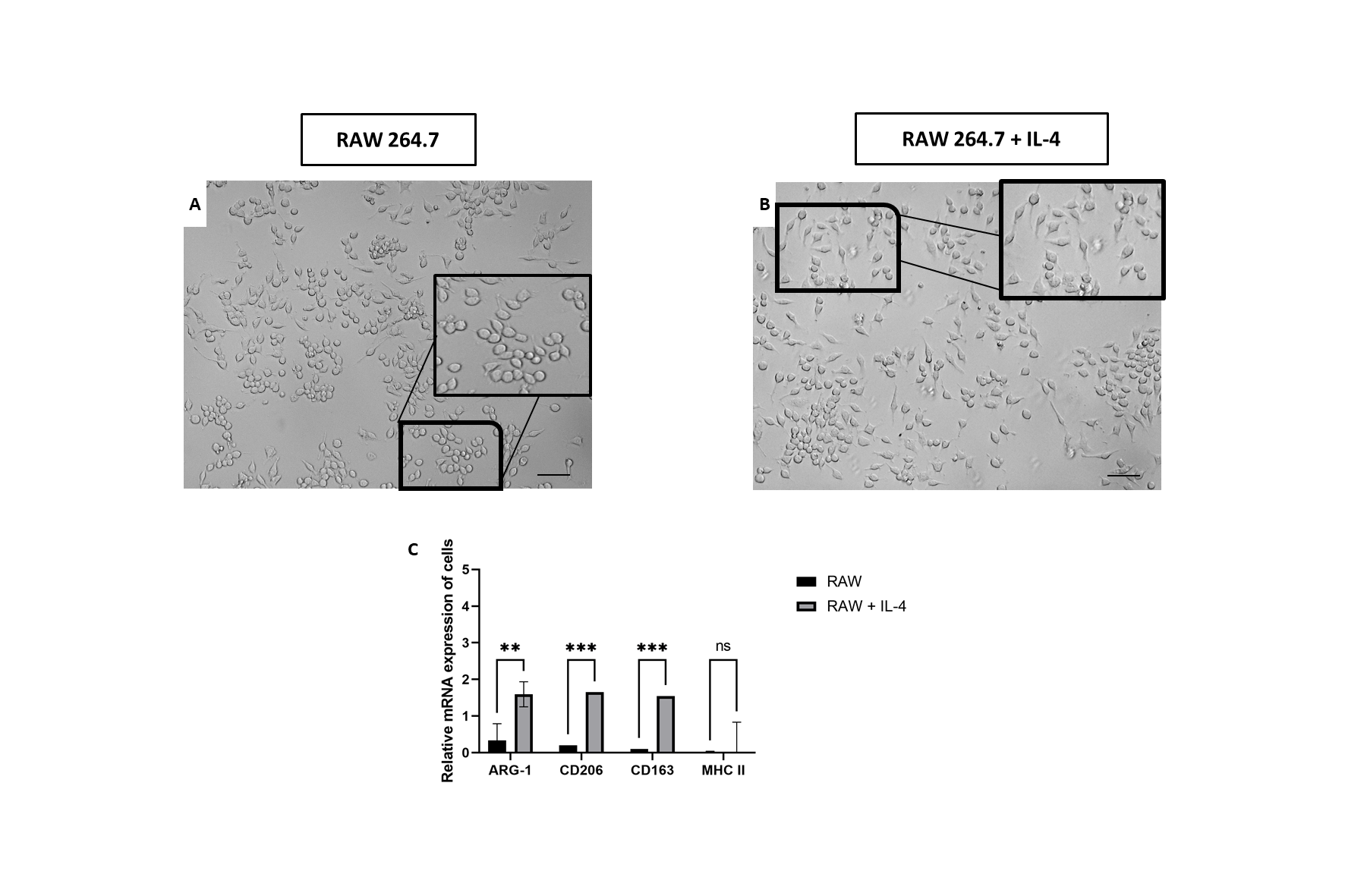

Supplement: Supplementary file 1 — Figure S1 1. [file MC-64-1450-s002.tif]

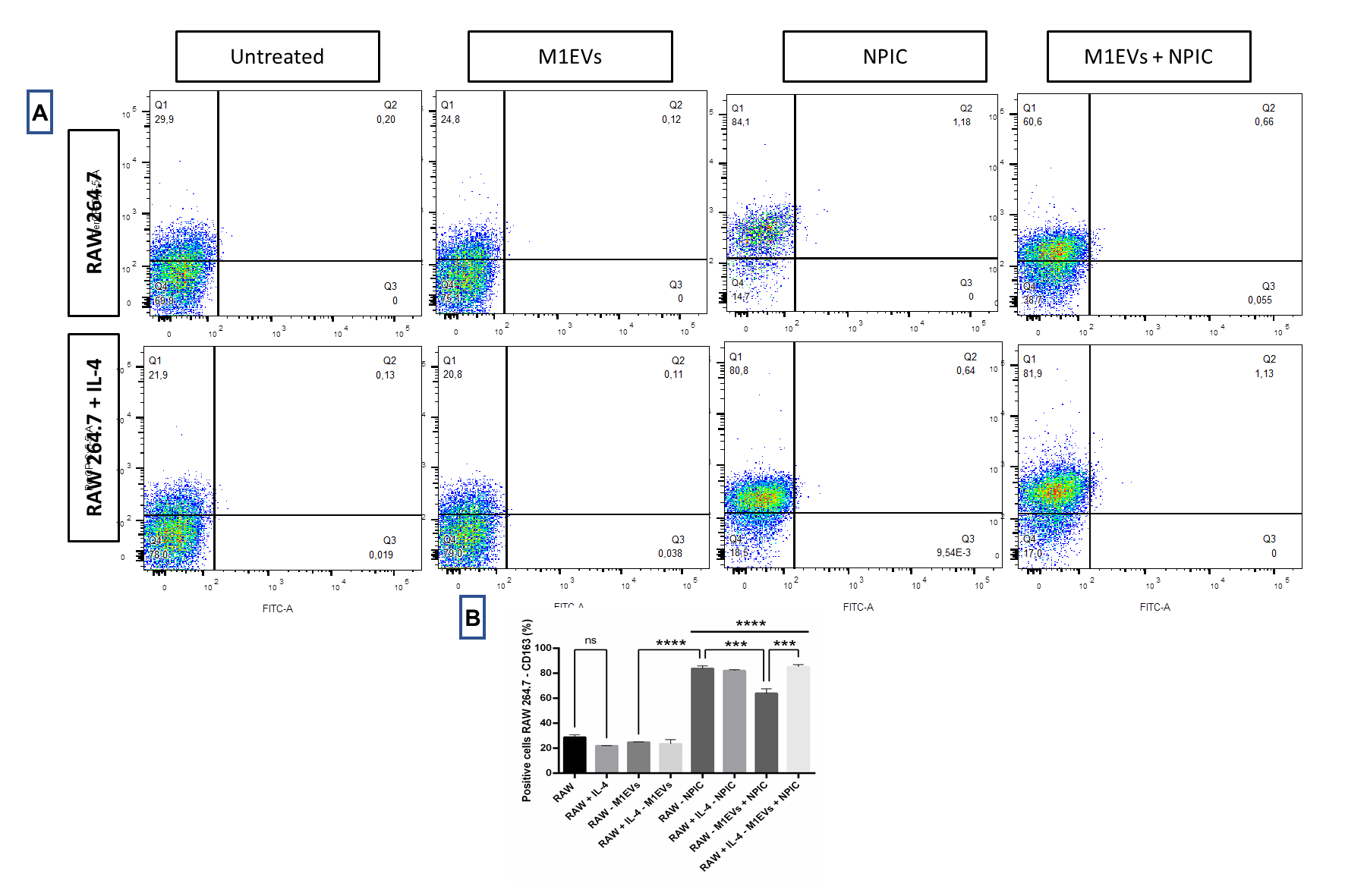

Supplement: Supplementary file 2 — Figure S2 1. [file MC-64-1450-s004.tif]

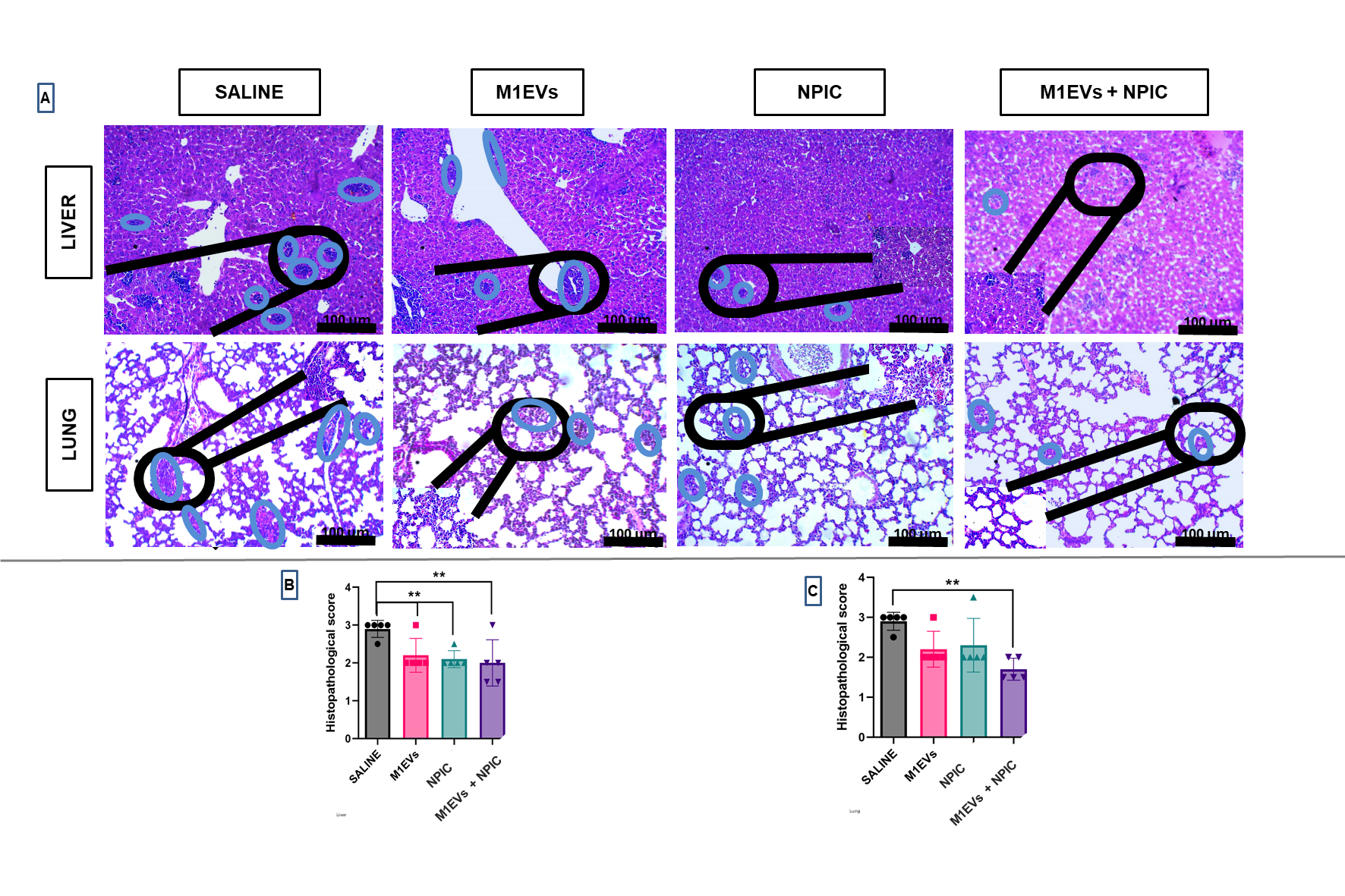

Supplement: Supplementary file 3 — Figure S3 1. [file MC-64-1450-s005.tif]

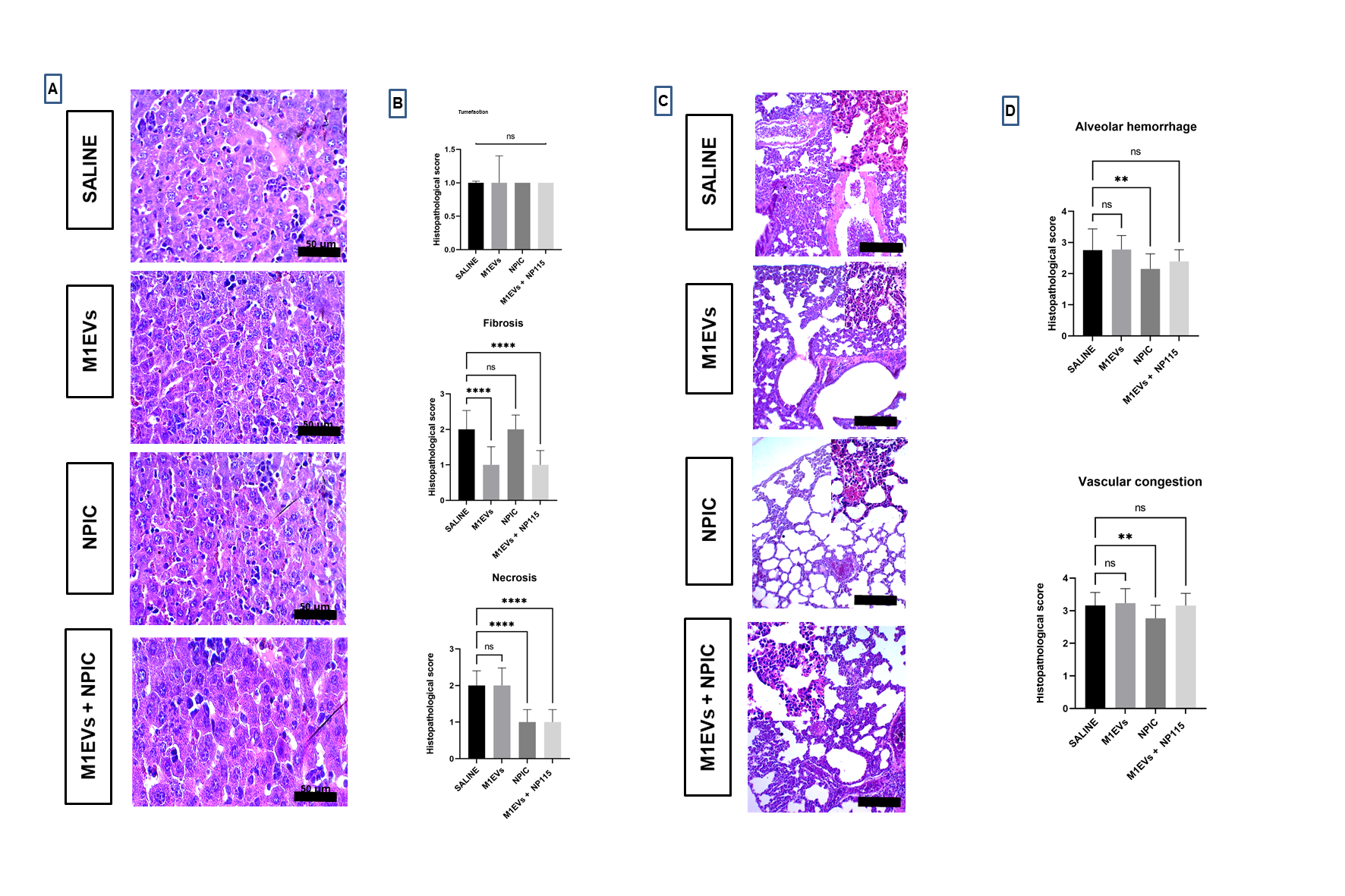

Supplement: Supplementary file 4 — Figure S4. [file MC-64-1450-s001.tif]
