# Supplementary material for: M1 Macrophage Extracellular Vesicles and TLR3 Agonist Nanoparticles Down‐Regulate Immunosuppression and Metastasis via AKT/TAM in Triple‐Negative Breast Cancer
Source: Mol Carcinog. 2025 Jun 24;64(9):1450–61. doi: 10.1002/mc.70003 (PMC12370002; doi:10.1002/mc.70003)
Supplement: Supplementary file 5 — Supporting Information revised. [file MC-64-1450-s003.docx]

**Supporting Information**

**Table 1.** Primer sequences for gene expression analysis of polarization of RAW 264.7 macrophage cells toward M2.

| **Gene** | **Primer Forward (F)** | **Primer Reverse (R)** | **Annealing temperature (°C)** |
| --- | --- | --- | --- |
| **β-actin** | AGGCCAACCTGTAAAAGATG | TGTGGTACGAGAGGCATAC | 50.94 |
| **ARG-1** | TGAGGAAAGCTGGTCTGCTG | ATCACCTTGCCAATCCCCAG | 54.79 |
| **CD163** | GGATCTCCGGGATGCTTCTG | CGCCTGCCAGACGAATATCT | 54.97 |
| **CD206** | GAGCCTGGAAAGAGCTGTGT | ACCCTCCGGTACTACAGCAT | 55.04 |
| **MHC II** | TGCTACTTCACCAACGGGAC | CGATGCCGCTCAACATCTTG | 54.76 |

**Table 2.** Primer sequences for gene expression analysis of tumor and lymph node.

| **Gene** | **Primer Forward (F)** | **Primer Reverse (R)** | **Annealing temperature (°C)** |
| --- | --- | --- | --- |
| **β-actin** | AGGCCAACCTGTAAAAGATG | TGTGGTACGAGAGGCATAC | 50.94 |
| **CD8** | GCTCAGTCATCAGCAACTCG | ATCACAGGCGAAGTCCAATC | 58 |
| **CD163** | GGATCTCCGGGATGCTTCTG | CGCCTGCCAGACGAATATCT | 54.97 |
| **CD80** | ACAACAGCCTTACCTTCGGG | CCATAGTTTTCCCCACCCCC | 60 |
| **AKT** | AGCGGCCGCAGGATGT | ACTGCGCCACAGAGAAGTTGT | 56 |
| **CD11c** | TTGTGGTCCTACTGTGCACC | CGAATGATGCTTGCAGCCTC | 54 |

**Table 3.** Reagents used for immunohistochemistry.

| **Antibody/Reagent** | **Catalog Number + Manufacturer** | **Dilution** | **Incubation Conditions** |
| --- | --- | --- | --- |
| Anti-PDL1 | Cat#66248-1-Ig, Proteintech | 1:200 | Overnight at 4°C |
| Anti-AKT | Cat#sc-5298, Santa Cruz Biotechnology | 1:100 | Overnight at 4°C |
| Anti-E-cadherin | Cat#14-3249-82, Invitrogen | 1:100 | Overnight at 4°C |
| Anti-CD8a | Cat#14-0081-82, Life Technologies Brasil | 1:400 | Overnight at 4°C |
| Anti-CD11c | Cat#13-0114-82, Life Technologies Brasil | 1:400 | Overnight at 4°C |
| Goat anti-mouse secondary antibody | Cat#31431, Invitrogen | 1:400 | Overnight at 4°C (for anti-AKT, anti-CD8a, anti-CD11c) |
| Polink2 HRP | GBI Labs | - | For anti-E-cadherin and anti-PDL1 |
| Diaminobenzidine (DAB) | Cat#EP-12-20542, EasyPath | - | Immunostaining detection |

**Table 4.** Mean red blood cell (RBC) counts and hematocrit values in the groups. Data are expressed as mean ± standard deviation (SD).

| **Group** | **RBC (10⁶/mm³)** | **Hematocrit (%)** |
| --- | --- | --- |
| **Saline** | **8.43 ± 1.29** | **34.75 ± 7.80** |
| **M1EVs** | **10.00 ± 2.35** | **42.25 ± 4.50** |
| **NPIC** | **8.19 ± 0.98** | **37.80 ± 4.44** |
| **M1EVs + NPIC** | **7.97 ± 0.54** | **43.00 ± 2.45** |

**Supplementary figure legends**

**Figure S1.** IL-4 induced the polarization of RAW 264.7 toward M2 macrophage. (A) RAW 264.7 are more oval cells, with few extensions (200X) and in the black rectangle (400X). (B) In RAW 264.7 + IL-4, larger cells are seen, with more extensions and taking up more space (200X) and in the black rectangle (400X). (C) Higher gene expression of ARG-1, CD206, CD163, but not MHCII . Data are shown as mean ± SD (**p < 0.01, ***p < 0.001 vs. control; ns: no significance). Scale bar: 50 μm.

**Figure S2.** Flow cytometry analysis of CD163 expression in RAW 264.7 and RAW 264.7 + IL-4 cells. In the panel A show CD163-positive cells in both cell types treated with saline (untreated), M1EVs, NPIC, and M1EVs + NPIC. The quantification is shown in panel B, indicating the percentage of CD163-positive cells for each treatment group. Data are presented as mean ± SD (***p < 0.001, ****p < 0.0001 vs. untreated; ***p < 0.001, ****p < 0.0001 between specific groups; ns: no significance). Statistical analysis was performed using two-way ANOVA followed by Kruskal-Wallis post hoc test..

**Figure S3.** Histopathological analysis of metastatic niches. Representative images of liver and lung parenchyma (100×) from animals treated with saline, M1EVs, NPIC, and M1EVs + NPIC are shown in panel A. Metastatic areas are indicated by blue circles. Panels B and C show the quantification of metastatic regions in the liver and lung, respectively. Data are expressed as mean ± SD (**p < 0.01 vs. saline; ns: no significance). Scale bar: 100 μm.

**Figure S4.** Toxicity evaluation through histopathological analysis of liver and lung tissues. Image A shows a representative liver section 400X, highlighting cellular alterations: tumefaction, fibrosis, and necrosis. The histopathological scores are presented in image B. Image C displays representative lung sections 400X and 100X, and image D shows the evaluation of alveolar hemorrhage and vascular congestion scores. Data are presented as mean ± SD. Statistical significance (**p < 0.01; ****p < 0.0001*;* ns = ns: no significance). Scale bar: 50 μm.
